# Iodized Salt Coverage and Influencing Factors in Chinese Out-of-Home Dining Venues: A Large Cross-Sectional Study from 31 Provinces of China

**DOI:** 10.3390/nu17152415

**Published:** 2025-07-24

**Authors:** Ying Zhang, Wei Ma, Jianqiang Wang, Haiyan Wang, Xiuwei Li, Jinpeng Wang, Jing Xu

**Affiliations:** 1 Key Laboratory of Public Nutrition and Health, National Health Commission of the Peoples’ Republic of China, Beijing 100050, China; zhangying@ninh.chinacdc.cn (Y.Z.);; 2 National Institute for Nutrition and Health, Chinese Center for Disease Control and Prevention, Beijing 100050, China

**Keywords:** iodized salt, out-of-home dining venues, coverage rate of iodized salt (CRIS), utilization rate of adequately iodized salt (URAIS), influencing factors

## Abstract

**Background/Objectives:** With the rising trend of out-of-home dining in China, the use of iodized salt (IS) in eating-out venues plays a key role in preventing iodine deficiency disorders (IDDs). However, the coverage rate of iodized salt (CRIS) and the utilization rate of adequately iodized salt (URAIS) in these venues in China remain underexplored, potentially undermining IDD prevention strategies. This study aims to assess the CRIS and URAIS in such venues across China and identify the factors influencing their prevalence. **Methods**: From 2021 to 2024, a nationwide cross-sectional study was conducted in China, involving 19,346 venues. A 50 g sample of cooking salt was collected from each venue, and the iodine content was measured. The CRIS and URAIS were calculated, and associations with various factors were assessed using Chi-square tests, the Cochran–Armitage trend test, and multivariate logistic regression. **Results**: Of the 19,346 samples, 18,519 tested positive for IS, and 17,588 contained adequately iodized salt (AIS), resulting in a CRIS of 95.7% and a URAIS of 90.9%. Significant regional differences were found, with coastal areas showing a lower CRIS and URAIS than inland areas (87.0% vs. 97.8%; 81.0% vs. 93.2%) and urbanized areas having lower rates compared to less urbanized areas (94.1% vs. 97.3%; 88.9% vs. 92.9%). Higher per capita income was associated with a lower CRIS and URAIS (Z = −19.72, *p* < 0.0001; Z = −13.85, *p* < 0.0001). Lower per capita income (OR = 3.24, OR = 1.36, *p* < 0.0001), inland areas (OR = 4.14, OR = 2.68, *p* < 0.0001), and mountainous areas (OR = 2.48, OR = 1.27, *p* < 0.0001) were associated with a higher likelihood of IS and AIS use. **Conclusions**: While the CRIS and URAIS in dining venues meet national standards, regional disparities persist, particularly in coastal, plain, and economically advanced areas. Strengthening regulatory oversight and public education on iodized salt’s health benefits is essential.

## 1. Introduction

Iodine is a crucial component of thyroid hormones, thyroxine (T4) and triiodothyronine (T3), and plays a pivotal role in human growth, development, and metabolic regulation [1]. Prolonged iodine insufficiency leads to iodine deficiency disorders (IDDs), primarily manifesting as endemic goiter, cretinism, and hypothyroidism [2]. IDDs remain a significant global public health challenge, with 1.9 billion individuals affected by iodine deficiency, including 285 million school-aged children [3]. In the past, China was one of the world’s most iodine-deficient regions. However, the implementation of the Universal Salt Iodization (USI) program in 1994 drastically reduced the prevalence of iodine deficiency. By 2013, China had achieved median urinary iodine concentrations (UICs) of 226.5 μg/L in children and stabilized goiter prevalence at 0.35% [2]. The “Criteria for elimination of iodine deficiency disorders (GB 16006-2008)” sets the criteria for iodine deficiency disease elimination, including a coverage rate of iodized salt (CRIS) ≥ 95% and a utilization rate of adequately iodized salt (URAIS) > 90% [4].

In recent years, rising income levels, urbanization, and rural development have increased dining out among Chinese residents. The proportion of individuals aged six and over dining out has increased from 14.6% in 2002 to 46.3% in 2017 [5]. Moreover, the rise of internet-based platforms has facilitated the growth of the catering industry, further boosting the frequency of dining out. Consequently, the contribution of household iodized salt to daily iodine intake has decreased [6]. A study by Song et al. found no statistically significant difference in urinary iodine levels between residents in areas with a URAIS ≥ 90% and those with a URAIS < 90%, suggesting that high dining-out frequency may play a role in iodine intake discrepancies [7]. The current monitoring of iodine deficiency in China relies on household salt consumption data, which does not account for iodized salt (IS) or adequately iodized salt (AIS) used in dining venues. Furthermore, the “Regulation on Salt Iodization to Reduce the Risk of Iodine Deficiency” only mandates using iodized salt in food products produced and sold in iodine-deficient areas, leaving gaps in guidelines for its use in restaurants.

An investigation of iodine use in dining venues was conducted to address these issues. In 2021–2022, a survey of 7889 eating establishments across 13 provinces showed that the CRIS in these venues was 95.2%, and the URAIS was 90.2%, with higher levels in inland areas than in coastal regions [8]. However, this survey was not nationally representative and lacked an in-depth analysis of factors influencing IS use. By the end of 2024, a broader sample of 19,346 dining venues from all 31 provinces was selected for salt iodine content. The need for comprehensive data is crucial. This research provides a comprehensive assessment of the prevalence of IS and AIS in dining venues nationwide and compares regional and economic differences, thereby enhancing our understanding of the factors affecting the CRIS and URAIS.

## 2. Materials and Methods

### 2.1. Study Design

This cross-sectional study, meticulously conducted from 2021 to 2024, spanned across 31 provinces in China. We used a multi-stage sampling method to select dining venues, ensuring a comprehensive representation of the national dining landscape. In the first stage of sampling, each province was divided into five geographical regions: east, south, west, north, and central. Then, two counties were randomly selected from each region using simple random sampling. In the second stage, each county was further divided into five sampling areas based on geographical orientation. Then, one township or sub-district with low iodine levels in drinking water was randomly selected in each sampling area using simple random sampling. Moreover, in the third stage, dining venues in each township or sub-district were divided into three strata: canteens, medium-sized restaurants, and small restaurants. A stratified sampling method was used to randomly select two canteens, five medium-sized restaurants, and five small restaurants.

### 2.2. Inclusion Criteria for Dining Venues

Dining venues were selected based on the “Industrial classification for national economic activities (GB/T 4754-2017)” [9] and included (1) restaurants categorized under 6210 (table meal service) and 6220 (fast-food service); (2) medium-sized restaurants, with a business area of 150–500 m^2^ or a seating capacity of 75–250 seats; (3) small restaurants, with a business area of less than 150 m^2^ or a seating capacity of fewer than 75 seats; and (4) institutional canteens (e.g., in schools, enterprises, and public institutions for internal workers and students). Only one chain restaurant per brand in each province was included.

### 2.3. Variables

Basic information about each surveyed area was collected, including whether the area is coastal or island, the geographic type (plains, mountains, hills), the population size, the number of people involved in agriculture, the per capita annual income, the types of venues, and the types of services. The urbanization rate was calculated using the following formula: Urbanization Rate = (Number of non-agricultural residents/Total population) × 100%. Counties were categorized into high- or low-urbanization areas based on the median urbanization rate. The counties were classified into high-income, middle-income, and low-income areas based on the per capita income. Types of venues included institutional canteens, medium-sized restaurants (MSRs), and small restaurants (SRs). Each venue’s service type was classified into either table meal service or fast-food service.

### 2.4. Sample Collection and Laboratory Testing

A 50 g sample of cooking salt was collected from each venue in a dry, clean plastic bag and sent to the laboratory for iodine content testing. The iodine content in salt (SIC) was measured according to the “General Test Method for Salt Industry (GB/T 13025.7-2012)” [10]. Iodized salt with KIO_3_ was analyzed through direct titration, while iodized salt with KI or other substances was tested using redox titration.

### 2.5. Standard of Iodine Content in Edible Salt

Salt with an SIC below 5 mg/kg was classified as non-iodized salt (NIS). AIS was defined as having an SIC within an acceptable range, with a deviation of ±30% from the average iodine content. “The iodine content of edible salt in the National Standard for Food Safety (GB26878-2011)” [11] specifies the iodine content in salt selected by 31 provinces in China. The average salt iodine content and the range of AIS in each province are shown in Table 1. Unqualified iodized salt included highly iodized salt (SIC above the upper limit) and low-iodized salt (SIC below the lower limit). The CRIS refers to the proportion of IS samples relative to the total samples tested, while the URAIS refers to the proportion of AIS samples relative to the total samples tested. The qualified rate of iodized salt (QRIS) was defined as the ratio of AIS samples to IS samples.

### 2.6. Statistical Analysis

Data entry was performed using Excel 2016, and statistical analysis was conducted using SAS version 9.4 (SAS Institute). Continuous variables are expressed as means ± standard deviation or medians (P25, P75), and categorical variables are presented as counts or percentages. For non-ordered categorical variables, including the region (coastal or island), terrain (plain, mountainous, or hilly), urbanization rate (high or low), type of venue (canteen, medium-sized restaurant, or small restaurant), and service type (table meal or fast food), the Chi-square test was used to compare whether there were statistical differences across these subgroups in the CRIS and URAIS. For ordered categorical variables, such as the per capita income level (high, medium, or low), the Cochran–Armitage trend test was used. Based on the results of the univariate tests, variables that showed statistical significance were selected as independent variables and included in a multivariable logistic regression model. In this model, the CRIS and URAIS were treated as dependent variables to identify the factors influencing these outcomes. All tests were two-sided; a *p*-value < 0.05 was considered statistically significant.

## 3. Results

### 3.1. Basic Information on Out-of-Home Dining Venues

The survey, which was comprehensive in its scope, covered 31 provinces, autonomous regions, and municipalities across China, encompassing 322 counties and a total population of 217.6 million people. The urbanization rate was 62.8%, and the annual per capita income was CNY 39.8 thousand. Of the 322 counties, 138 were in plains, 104 in mountainous regions, and 80 in hilly areas. Coastal counties accounted for 18.9% (61 counties) of the sample, distributed across nine provinces: Liaoning, Shanghai, Jiangsu, Zhejiang, Fujian, Shandong, Guangdong, Guangxi, and Hainan. The survey achieved a remarkable 100% completion rate, with 19,346 dining venues surveyed. Among these, 3222 were institutional canteens, 7862 were medium-sized, and 8262 were small. Most venues (15,766) provided table meal service, while 3580 offered fast food (Table 2).

### 3.2. Iodized Salt Testing in Dining Venues

Salt samples from all 19,346 dining venues were tested, with 18,519 testing positive for iodized salt, yielding a CRIS of 95.7%. The CRIS in central and west regions was greater than 95%, with significant regional variation (χ^2^ = 729.37, *p* < 0.0001). In total, 17,588 samples contained adequately iodized salt, resulting in a URAIS of 90.9%. The URAIS was great than 90% in the central and west regions, with regional differences (χ^2^ = 485.22, *p* < 0.0001). The CRIS and URAIS by region are shown in Table 3.

**Figure 1:**
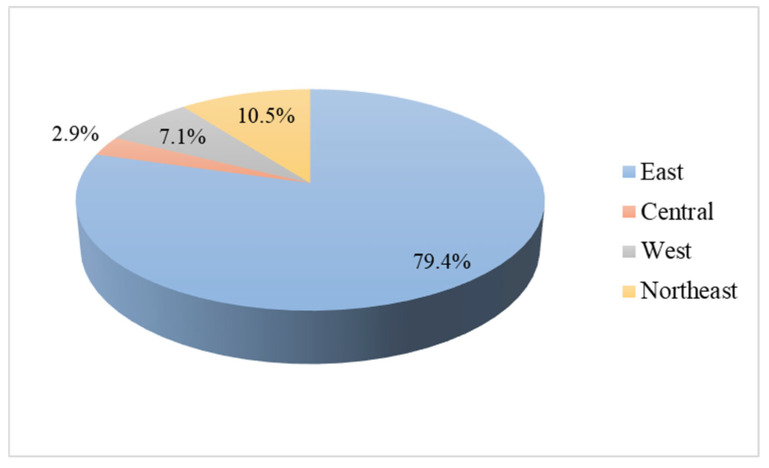
Distribution of non-iodized salt in out-of-home dining venues in in four regions.

**Figure 2:**
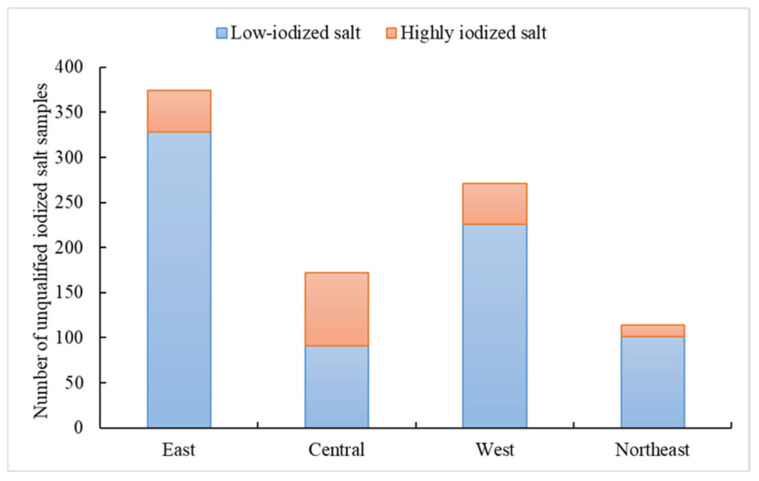
Distribution of unqualified iodized salt in out-of-home dining venues in in four regions.

A total of 827 samples were identified as NIS, primarily from east (79.4%)and northeast regions (10.5%) (Figure 1). Additionally, 931 samples of iodized salt were classified as unqualified, with 80.1% being low-iodized salt, mainly from east, west, and northeast region. The remaining 19.9% were highly iodized salt, predominantly from central region (Figure 2).

The median iodine content of the 18,519 IS samples was 25.2 mg/kg, ranging from 24.1 mg/kg in the northeast to 26.3 mg/kg in the west region. The coefficient of variation (CV) for iodine content across all samples was 16.2%, with the lowest CV in the west region (15.3%) and the highest in the northeast region (17.8%) (Table 3).

### 3.3. Influencing Factors of CRIS and URAIS in Dining Venues

Approximately 18.9% of the salt samples were from coastal areas, where the CRIS was 87.0%, significantly lower than the 97.8% observed in inland areas (χ^2^ = 845.71, *p* < 0.0001). The URAIS in coastal areas was 81.0%, also significantly lower than the 93.2% in inland areas (χ^2^ = 538.31, *p* < 0.0001). As shown in Table 4, 42.9% of salt samples were from plain regions, 32.3% from mountainous areas, and 24.8% from hilly areas. Both plain and hilly areas had a CRIS <95%, significantly lower than that of mountainous areas (χ^2^ = 245.79, *p* < 0.0001). Similarly, the URAIS in plains and hilly regions was <90%, significantly lower than that in mountainous regions (χ^2^ = 140.16, *p* < 0.0001).

Counties were categorized into high- and low-urbanization areas based on the median urbanization rate. In areas with a high urbanization rate, 49.9% of salt samples were collected, and the CRIS was 94.1%, significantly lower than the 97.3% observed in low-urbanization areas (χ^2^ = 121.61, *p* < 0.0001). The URAIS in high-urbanization areas was 88.9%, significantly lower than the 92.9% in low-urbanization areas (χ^2^ = 96.50, *p* < 0.0001). The counties were classified into high-income, middle-income, and low-income areas based on the per capita income. As the income levels increased, both the CRIS (Z = −19.72, *p* < 0.0001) and URAIS (Z = −13.85, *p* < 0.0001) decreased significantly (Table 4).

Regarding the type of dining venue, 16.7% of the salt samples came from canteens, 40.6% from medium-sized restaurants, and 42.7% from small restaurants. The CRIS in canteens was 94.7%, significantly lower than that in medium-sized and small restaurants (χ^2^ = 10.12, *p* = 0.0063). The URAIS was greater than 90% in all dining venues, with no significant subgroup differences (χ^2^ = 2.11, *p* = 0.35). Among the dining venues, 81.5% provided table meal service, and both types of establishments (table meal and fast food) had a CRIS >95%, with no significant subgroup differences (χ^2^ = 0.31, *p* = 0.58). Similarly, the URAIS was greater than 90% in both types, with no significant difference (χ^2^ = 2.13, *p* = 0.14) (Table 4).

### 3.4. Multivariable Logistic Regression Analysis of CRIS and URAIS

Unconditional logistic regression was performed on the five statistically significant factors identified in the univariate analysis, with the CRIS as the dependent variable. The regression results indicated that coastal versus inland areas, the geographic type, the per capita income level, and the type of venue significantly affected the CRIS (*p* < 0.01). In areas with a low per capita income and medium-income regions, the likelihood of using IS was 3.24 times (OR = 3.24, 95% CI: 2.52–4.15) and 2.04 times (OR = 2.04, 95% CI: 1.73–2.42) higher, respectively, compared to in high-income areas. Inland areas were 4.14 times more likely to use IS than coastal areas (OR = 4.14, 95% CI: 3.56–4.83), while mountainous areas were 2.48 times more likely to use IS than plains (OR = 2.48, 95% CI: 1.86–3.30). Medium-sized and small restaurants were 1.38 (OR = 1.38, 95% CI: 1.13–1.68) and 1.30 (OR = 1.30, 95% CI: 1.07–1.58) times more likely to use IS than canteens (Table 5).

Using the URAIS as the dependent variable, unconditional logistic regression analysis was performed on four statistically significant factors from the univariate analysis. The results showed that the urbanization rate, the per capita income, coastal versus inland areas, and the geographic type significantly affected the URAIS (*p* < 0.01). Areas with low urbanization rates were 1.22 times more likely to use AIS than high-urbanization areas (OR = 1.22, 95% CI: 1.08–1.38). The likelihood of using AIS was 1.36 times higher in low-income areas and 1.27 times higher in medium-income areas than in high-income areas (OR = 1.36, 95% CI: 1.16–1.59; OR = 1.27, 95% CI: 1.12–1.44). Inland areas were 2.68 times more likely to use AIS than coastal areas (OR = 2.68, 95% CI: 2.40–3.00), and mountainous areas were 1.27 times more likely to use AIS than plain areas (OR = 1.27, 95% CI: 1.10–1.47) (Table 5).

## 4. Discussion

This national survey provided a comprehensive assessment of IS and AIS usage in dining venues across 31 provinces of China. The findings, which revealed significant regional disparities, underscore the urgent need for targeted interventions. While the high overall CRIS (95.7%) and URAIS (90.9%) are encouraging, the disparities in coastal regions and areas with higher urbanization rates and income levels are a cause for concern. Dining venues in plains and hilly regions showed a lower CRIS and URAIS than those in mountainous areas. The variations in iodine content across provinces and different types of dining venues, with institutional canteens generally exhibiting a lower CRIS than restaurants, further highlight the importance of these interventions.

Iodine is an essential nutrient for thyroid hormone production, and insufficient intake can lead to IDDs, such as goiter, impaired thyroid function, growth retardation, and low intelligence. China used to be one of the most serious iodine deficiency countries in the world. According to a survey in the 1970s in China, the iodine deficiency area covered a population of 370 million, with 35 million endemic goiter patients and 250,000 patients with typical cretinism [12]. In the 1990s, about 720 million people were living in iodine-deficient areas in the country, with 8 million people suffering from visible goiter and 180,000 patients with cretinism [13]. Since the implementation of the USI in 1994, China has made significant achievements in the control and elimination of IDDs. The results of the fourth National Iodine Deficiency Disease Monitoring in 2002 showed that China has almost eliminated IDDs [14]. National monitoring in 2022 [15] revealed that the median urinary iodine level in children aged 8 to 10 years was 212.4 μg/L, the proportion of urinary iodine less than 50 μg/L was 3.1%, and the rate of goiter in children nationwide was 1.5%. The indicators met the requirements of IDD elimination standards at the national level. USI has proven to be an effective and safe strategy for controlling IDDs. In line with the monitoring indicators recommended by the WHO/UNICEF/ICCIDD [16], China established the “China Iodine Deficiency Disease Elimination Standard (GB 16006-2008)” in 2008 [4], which set the household CRIS ≥ 95% and URAIS > 90% as benchmarks for eliminating IDDs. The results of the 2022 National Household Salt Iodization Monitoring [15] indicated that the national household CRIS was 95.6%, which is essentially the same as the CRIS in dining venues (95.7%) in this study. The URAIS in households nationwide was 91.2%, slightly higher than that in dining venues (90.9%). In some provinces in the eastern part of the country, CRIS was less than 95% in both households and dining venues. The above comparisons suggest a notable similarity between the use of iodized salt in both households and out-of-home dining venues. This finding underscores the importance of ongoing efforts to enhance both the CRIS and URAIS, not only in households but also in dining venues, particularly in provinces where the use of iodized salt remains suboptimal.

In developed countries, up to 75% of total salt intake comes from foods consumed outside the home or processed foods [17]. Most studies have focused on using iodized salt in processed foods [18], with fewer investigating its use in restaurants. A study in Cape Verde found that 97% of restaurants and bakeries used iodized salt [17], while a study in the United States reported that 65% of sit-down restaurants and 32% of fast-food restaurants used iodized salt [19]. As the global consumption of processed foods and dining out continues to rise, household salt consumption is decreasing as a proportion of the total salt intake [20]. In recent years, with the rapid development of China’s economy and the improvement in people’s living standards, an increasing number of residents are opting to eat out. The China Health and Nutrition Survey revealed that the rate of Chinese residents eating out increased from 46.0% to 57.6% between 2000 and 2018. Specifically, the rate of urban residents eating out decreased slightly, from 76.9% to 71.6%, while the rate of rural residents eating out increased markedly, from 30.2% to 47.8% [21]. The Nutrition and Health status of Chinese residents from 2015 to 2017 showed that the average daily cooking salt intake among residents was 9.3 g. The intake in urban and rural areas was 8.9 g and 9.6 g, respectively [22]. The study by Li Li et al. [23] showed that the median amount of salt used in restaurants in six provinces of China was 5.8 g/person, which provided approximately 145 μg of iodine per person. The study by Li et al. [24] showed that the median amount of salt consumed per person in restaurants in seven provinces of China was 3.52 g per person, which provided approximately 88 μg of iodine per person. In summary, the iodine consumed through eating out has a significant impact on the population’s iodine nutritional status. Therefore, understanding the use of iodized salt in out-of-home dining venues is essential for evaluating the iodine nutritional status of populations and informing relevant public health policies.

A previous study [8] investigated iodized salt use in 7889 dining establishments across 13 provinces. We found that the CRIS and URAIS in coastal venues were significantly lower than those in inland establishments. To further confirm this regional disparity, we expanded our sample size in the current study to include 19,346 dining venues across 31 provinces. The results showed that the CRIS and URAIS in 3661 coastal venues were significantly lower than those in 15,685 inland venues, consistent with household iodized salt consumption patterns. A survey in Zhejiang Province revealed that the URAIS was substantially lower in coastal households than inland areas (33.9% vs. 77.6%) [25]. Similarly, a cross-sectional survey conducted by Qian Tingting et al. in 2020 across 28 regions in seven provinces found that coastal areas had a lower CRIS (88.2%) and URAIS (84.7%) compared to inland areas, where the rates were 98.0% and 96.3%, respectively [26]. Interviews with operators of dining venues in coastal regions revealed common misconceptions, such as the belief that seafood, commonly consumed in these areas, obviates the need for iodized salt. Some operators also neglected to check whether the salt they purchased was iodized. Additionally, the presence of private salt farms in coastal regions likely contributes to the use of non-iodized sea salt, further reducing iodized salt coverage. Coastal residents often consume more seafood, which contains slightly more iodine than land-based animal products, but these foods are typically prepared without added salt, resulting in lower iodine intake. While seaweed, such as kelp, is the highest iodine source among seafood, its consumption is limited. Given these findings, we recommend strengthening public awareness campaigns targeting dining establishments, particularly in coastal regions, to educate operators and procurement personnel on the importance of iodized salt in preventing IDDs. Ensuring that iodized salt is consciously purchased and used will help improve iodine intake and support public health goals.

This study presents a novel finding: as urbanization and income levels increase, the CRIS and URAIS in dining establishments show a decreasing trend. This phenomenon has not been directly observed or reported in the existing Chinese literature. Household iodized salt monitoring data reveal that the CRIS and URAIS are lower in more urbanized and economically developed regions. For instance, monitoring data from 2019 to 2022 indicate that in big cities like Shanghai, Tianjin, and Beijing, the URAIS often falls below 90% [15,27,28,29], primarily due to the widespread circulation of non-iodized table salt. Several factors may explain this trend. First, demographic and health awareness differences may play a role. Urban areas attract highly educated, high-income individuals with greater scientific information access. These high-income individuals are more health-conscious and may proactively reduce their iodized salt intake based on medical check-ups indicating adequate iodine levels. Survey data [30] suggest that 34.2% of residents in large cities avoid iodized salt because they believe they are not iodine-deficient. In comparison, 18.6% choose non-iodized salt, citing concerns over its health implications, especially about excessive iodine intake and thyroid diseases. Second, disparities in public service resource allocation are significant. The concentration of healthcare resources in urban centers and in high-income populations facilitates easier access to thyroid screening services, leading to the increased detection of thyroid disorders such as hyperthyroidism and thyroid nodules. These conditions are often associated with excessive iodine intake, which, despite the lack of direct evidence linking iodized salt to thyroid cancer, has been amplified through media reports and health campaigns, influencing dietary choices [31]. Third, market diversification contributes to this trend. In economically advanced regions, supermarkets and e-commerce platforms offer a wide variety of non-iodized salts (e.g., rock salt, sea salt), catering to consumer preferences for “natural” and “healthier” products. Although iodized salt is typically cheaper than non-iodized alternatives, higher-income groups, being less price-sensitive, are willing to pay a premium for “natural” and “healthier” non-iodized salt. In contrast, international studies from low-income developing countries, such as Pakistan and Ethiopia [32,33], have shown that iodized salt coverage increases as income levels rise. In these regions, however, low-income households face several barriers to iodized salt use, including economic constraints (with iodized salt often being more expensive) [33], limited health education and awareness [34], and an unstable supply of iodized salt [35]. These factors result in a lower usage rate among low-income groups. The differing trends in the relationship between income levels and iodized salt coverage, both domestically and internationally, may reflect variations in iodization policies, salt price structures, and the overall health literacy of the population in different countries. This analysis underscores the complex interplay of socio-economic, demographic, and market factors influencing iodized salt consumption, highlighting the need for tailored public health strategies in urbanized and economically developed regions.

This study also reveals a significant regional variation in the CRIS and URAIS, with mountainous areas showing higher rates than plains and hilly regions in eating-out places. This disparity is primarily driven by the more severe natural iodine deficiency in mountainous areas, leading to a greater public health focus on preventing iodine deficiency. Epidemiological surveys indicate that the geographic distribution of iodine deficiency diseases in China follows a pattern of “mountains > hills > plains > coasts” [36]. Mountainous and remote regions typically have lower iodine content in soil and water, which results in insufficient iodine intake through food and drinking water, leading to higher risks of iodine deficiency diseases. In contrast, coastal and plain areas benefit from higher iodine levels in the environment, making it easier for residents to obtain adequate iodine through their diets. This regional variation has prompted targeted public health interventions, particularly in the west region, which were classified as “difficult areas” for iodine deficiency disease elimination [37]. The government implemented financial subsidies, direct iodized salt distribution to households, and a vertical management system to improve iodized salt coverage in these regions [38]. Additionally, the dietary habits of residents in mountainous areas, which are more homogeneous and reliant on local agricultural products with minimal seafood intake, further reinforce their dependence on iodized salt, resulting in higher usage rates. In contrast, residents in plains benefit from a more diverse diet, which provides alternative sources of iodine, leading to a lower reliance on iodized salt. These findings highlight how natural conditions and dietary structures influence iodized salt usage behaviors across different topographies and underscore the need for targeted policy interventions and resource allocation in iodine-deficient areas.

This study offers several notable strengths. First, it utilizes monitoring data from 19,346 out-of-home dining venues across 31 provinces in China, providing a large, nationally representative sample. This robust dataset fully reflects the actual situation of the CRIS and URAIS in dining establishments across various regions in China. Second, this study is the first to report that the CRIS and URAIS in eating-out venues are decreasing amidst urbanization and rising income levels. This finding, which has not been directly addressed in the existing literature, holds significant academic value. It offers new insights for developing iodized salt policy and provides a solid theoretical foundation for investigating regional differences in consumption behaviors. Lastly, the study employs a multifactorial analytical approach, considering not only economic factors like urbanization and income but also geographical influences such as coastal versus inland locations, topographical variations, and the types of dining venues. This comprehensive analysis allows a more nuanced understanding of the factors driving iodized salt consumption. However, the study also has certain limitations. Firstly, due to its cross-sectional design, causal relationships between the factors cannot be definitively established. Future longitudinal studies are needed to validate these findings further. Secondly, the study does not account for other potentially confounding variables, such as the purchase date and storage conditions of iodized salt or the level of awareness among restaurant operators. Prolonged storage and exposure to humidity or heat can lead to iodine loss, which may result in the misclassification of iodization adequacy. Future studies should systematically account for these factors to provide a more accurate estimation of iodine content and ensure more reliable assessments of iodized salt usage. Finally, this study was unable to perform a stratified analysis between independent restaurants and national or international chain restaurants, as our design limited each province to no more than one chain restaurant, with many provinces not including any chain establishments. In future research, we plan to incorporate independent and chain restaurants as stratification criteria and perform stratified sampling to explore potential differences between these two types of establishments.

## 5. Conclusions

This study highlights that while iodized salt coverage in the catering industry generally meets the required standards, significant regional disparities persist, raising public health concerns. In economically developed cities, coastal areas, and regions with higher-income populations, the CRIS and the URAIS in dining establishments are notably lower than the national average. These disparities suggest that certain regions are lagging in both the availability and quality of iodized salt in food service settings. To address these issues, it is crucial to enhance regulatory efforts in these regions, including conducting regular inspections and assessments of iodized salt use in dining venues. Local governments must also adopt more stringent enforcement measures to ensure compliance with iodization standards. Moreover, increasing public awareness about the health benefits of iodized salt is crucial to correcting misconceptions and mitigating the over-reliance on non-iodized salt in these regions.

## Figures and Tables

**Table 1:** The average iodine content and the range of AIS in each province in China.

Range of AIS (mg/kg)	Iodine Content in Salt (mg/kg)	Provinces/Autonomous Regions/Municipalities
18~33	25	Shaanxi, Hainan, Hubei, Guangxi, Jiangxi, Anhui, Yunnan, Shanxi, Jiangsu, Fujian, Inner Mongolia, Shandong, Zhejiang, Jilin
21~39	30	Sichuan, Gansu, Guizhou, Qinghai, Hunan, Chongqing, Henan, Ningxia, Tibet, Tianjin, Shanghai, Xinjiang
18~39	25 or 30	Heilongjiang, Liaoning, Hebei, Beijing, Guangdong

AIS, adequately iodized salt.

**Table 2:** Characteristics of the surveyed areas and out-of-home dining venues.

Regions	Counties (*N*)			Topography (*N*)			Residents (Million)	Urbanization Rate (%)	PCI (Thousand CNY/Year)	Dine-Out Venues (*N*)				Service Type (*N*)	
	Total	Coastal	Inland	Plain	Mountain	Hill				Total	Canteen	MSR	SR	Table Meal	Fast Food
East	121	56	65	73	22	56	102.7	72.8	50.5	7265	1213	3007	3045	5660	1605
Central	61	0	61	23	13	0	41.5	50.9	33.1	3671	622	1509	1540	3109	562
West	115	3	112	29	64	3	56.5	53.4	33.9	6933	1161	2722	3050	5753	1180
Northeast	25	2	23	13	5	2	16.9	62.2	31.8	1477	226	624	627	1244	233
Total	322	61	261	138	104	80	217.6	62.8	39.8	19,346	3222	7862	8262	15,766	3580

*N*, number; PCI, per capita annual income; CNY, Chinese Yuan; MSR, medium-sized restaurant; SR, small restaurant.

**Table 3:** Results of the survey on iodized salt in out-of-home dining venues in different regions.

Regions	IS					AIS		
	*N*	CRIS (%)	Mean ± SD (mg/kg)	Median (P25, P75) (mg/kg)	CV (%)	*N*	URAIS (%)	QRIS (%)
East	6608	91.0	24.3 ± 3.8	24.4 (22.3,26.4)	15.6	6234	85.8	94.3
Central	3647	99.4	25.4 ± 4.1	25.2 (22.7,27.8)	16.0	3475	94.7	95.3
West	6874	99.2	26.4 ± 4.1	26.3 (23.9,28.8)	15.3	6603	95.2	96.1
Northeast	1390	94.1	24.1 ± 4.3	24.1 (21.6,26.8)	17.8	1276	86.4	91.8
Total	18,519	95.7	25.3 ± 4.1	25.2 (22.8, 27.6)	16.2	17,588	90.9	95.0

*N*, number; IS, iodized salt; AIS, adequately iodized salt; CRIS, coverage rate of iodized salt; URAIS, utilization rate of adequately iodized salt; QRIS, qualified rate of iodized salt; CV, coefficient of variation; SD, standard deviation.

**Table 4:** Factors affecting iodized salt coverage and consumption of adequately iodized salt in dining venues.

Variables	Salt		IS				AIS			
	*N*	%	*N*	CRIS (%)	χ^2^	*p*	*N*	URAIS (%)	χ^2^	*p*
Region					845.71	<0.0001			538.31	<0.0001
Coastal	3661	18.9	3184	87.0			2965	81.0		
Inland	15,685	81.1	15,335	97.8			14,623	93.2		
Terrain					245.79	<0.0001			140.16	<0.0001
Plains	8297	42.9	7810	94.1 ^a^			7396	89.1 ^a^		
Mountains	6253	32.3	6192	99.0 ^b^			5906	94.5 ^b^		
Hills	4796	24.8	4517	94.2 ^a^			4286	89.4 ^a^		
Urbanization rate					121.61	<0.0001			96.50	<0.0001
High	9658	49.9	9090	94.1			8584	88.9		
Low	9688	50.1	9429	97.3			9004	92.9		
Per capita income					−19.72 ^c^	<0.0001			−13.85 ^c^	<0.0001
High	6437	33.3	5904	91.7			5603	87.0		
Medium	6408	33.1	6196	96.7			5871	91.6		
Low	6501	33.6	6419	98.7			6114	94.1		
Type of venue					10.12	0.0063			2.11	0.35
Canteen	3222	16.7	3051	94.7 ^b^			2908	90.3		
MSR	7862	40.6	7545	96.0 ^a^			7163	91.1		
SR	8262	42.7	7923	95.9 ^a^			7517	91.0		
Type of service					0.31	0.58			2.13	0.14
Table meal	15,766	81.5	15,086	95.7			14,356	91.1		
Fast food	3580	18.5	3433	95.9			3232	90.3		

IS, iodized salt; AIS, adequately iodized salt; CRIS, coverage rate of iodized salt; URAIS, utilization rate of adequately iodized salt. ^a^ The difference was not statistically significant. ^b^ The difference was statistically significant. ^c^ The Z-value of the Cochran–Armitage trend test.

**Table 5:** Multivariate analysis of the use of iodized salt and adequately iodized salt in out-of-home dining venues.

Variable	CRIS					URAIS				
	b	S_b_	Wald χ^2^	*p*	OR (95%CI)	b	S_b_	Wald χ2	*p*	OR (95%CI)
Intercept	1.41	0.09	223.93	<0.0001		1.30	0.05	644.68	<0.0001	
Urbanization rate (reference = high)										
Low						0.20	0.06	10.70	0.0011	1.22 (1.08, 1.38)
Per capita income level (reference = high)										
Medium	0.71	0.09	70.35	<0.0001	2.04 (1.73, 2.42)	0.24	0.06	13.86	0.0002	1.27 (1.12, 1.44)
Low	1.17	0.13	84.91	<0.0001	3.24 (2.52, 4.15)	0.30	0.08	13.84	0.0002	1.36 (1.16, 1.59)
Area (reference = coastal)										
Inland	1.42	0.08	334.64	<0.0001	4.14 (3.56, 4.83)	0.99	0.06	293.72	<0.0001	2.68 (2.40, 3.00)
Terrain (reference = plains)										
Mountainous	0.91	0.15	38.53	<0.0001	2.48 (1.86, 3.30)	0.24	0.07	10.83	0.0010	1.27 (1.10, 1.47)
Hilly	−0.26	0.08	10.39	0.0013	0.77 (0.66, 0.90)	−0.11	0.06	3.50	0.061	0.89 (0.79, 1.01)
Type of eating-out place (reference = canteen)										
MSR	0.32	0.10	10.03	0.0015	1.38 (1.13, 1.68)					
SR	0.26	0.10	7.07	0.0079	1.30 (1.07, 1.58)					

CRIS, coverage rate of iodized salt; URAIS, utilization rate of adequately iodized salt; MSR, medium-sized restaurant; SR, small restaurant; OR, Odds Ratio.

## Data Availability

The data presented in this study are available on request from the corresponding author due to privacy.

## Abbreviations

The following abbreviations are used in this manuscript:

IS	Iodized salt
AIS	Adequately iodized salt
NIS	Non-iodized salt
CRIS	Coverage rate of iodized salt
URAIS	Utilization rate of adequately iodized salt
QRIS	Qualified rate of iodized salt
UICs	Urinary iodine concentrations
SIC	Iodine content in salt
IDDs	Iodine deficiency disorders
USI	Universal Salt Iodization
*N*	Number
CV	Coefficient of variation
SD	Standard deviation
OR	Odds Ratio
MSRs	Medium-sized restaurants
SRs	Small restaurants
PCI	Per capita annual income
CNY	Chinese Yuan
